# ROS-mediated enhanced transcription of CYP38 promotes the plant tolerance to high light stress by suppressing GTPase activation of PsbO2

**DOI:** 10.3389/fpls.2015.00777

**Published:** 2015-09-29

**Authors:** Yongqiang Wang, Lizhang Zeng, Da Xing

**Affiliations:** MOE Key Laboratory of Laser Life Science and Institute of Laser Life Science, College of Biophotonics, South China Normal UniversityGuangzhou, China

**Keywords:** *Arabidopsis*, high light, CYP38, PsbO2, reactive oxygen species, D1 protein

## Abstract

As a member of the Immunophilin family, cyclophilin38 (CYP38) is discovered to be localized in the thylakoid lumen, and is reported to be a participant in the function regulation of thylakoid membrane protein. However, the molecule mechanisms remain unclear. We found that, CYP38 plays an important role in the process of regulating and protecting the plant to resist high light (HL) stress. Under HL condition, the gene expression of *CYP38* is enhanced, and if *CYP38* gene is deficient, photochemistry efficiency, and chlorophyll content falls distinctly, and excessive reactive oxygen species synthesis occurs in the chloroplast. Western blot results showed that the D1 degradation rate of *cyp38* mutant plants is faster than that of wide type plants. Interestingly, both gene expression and activity of PsbO2 were drastically enhanced in *cyp38* mutant plants and less changed when the deleted gene of CYP38 was restored under HL treatment. This indicates that CYP38 may impose a negative regulation effect on PsbO2, which exerts a positive regulation effect in facilitating the dephosphorylation and subsequent degradation of D1. It is also found that, under HL condition, the cytoplasmic calcium ([Ca^2+^]_cyt_) concentration and the gene expression level of *calmodulin 3* (*CaM3*) arose markedly, which occurs upstream of *CYP38* gene expression. In conclusion, our results indicate that CYP38 plays an important role in plant strengthening HL resistibility, which provides a new insight in the research of mechanisms of CYP38 protein in plants.

## Introduction

The light-dependent reactions and subsequent oxygen evolution in photosynthesis are performed by four multi-subunit protein complexes within the thylakoid membranes of chloroplast. They are Photosystem I (PSI) and Photosystem II (PSII), cytochrome *b_6_f* complex, and CFo–CF_1_ complex (Shi and Schröder, 2004; Barber, 2006; Nelson and Yocum, 2006). Among these multi-subunit protein complexes, PSII serves as a light-driven water-plastoquinone oxidoreductase in the thylakoid membranes of oxygenic photosynthetic organisms, included in the process of oxygen-evolution and a membrane-embedded light-harvesting chlorophyll a/b complex (LHCII) that directs the light energy to the PSII core. As the essence of photosynthesis, light also can lead to inactivation of PSII and causes damage to the D1 protein in the reaction center, especially triggered by high light (HL) conditions. Due to such light-induced damage, D1 turns over at a higher rate than all other thylakoid polypeptides (Mattoo et al., 1981). Photodamage to PSII and attendant turnover of D1 protein for PSII repairing occur at all degrees of light intensity. It requires a number of processes to repair photodamaged PSII, which include (i) phosphorylation of the damaged D1 protein and migration from the grana to the stroma membrane regions of thylakoid, (ii) dephosphorylation of p-D1 (p-D1 instead of phosphorylated D1), and then its degradation, (iii) reassembly of new D1 protein into functional PSII complexes (Adir et al., 1990; Barbato et al., 1992; Prasil et al., 1992; Aro et al., 1993; Rintamäki et al., 1996). However, these processes need much more precise molecular regulations which have not yet been completely unveiled.

Initially, immunophilins are found to be cellular receptor proteins for immunosuppressive drugs: FK506 and cyclosporin A (Schreiber, 1991). The receptor proteins for FK506 and cyclosporin A, respectively, called FK506-binding proteins (FKBPs) and cyclophilins (CYPs), are indicated as immunophilins collectively. Now it is known that these proteins widely exist in organisms, from bacteria and fungi to animals and plants. Genes encoding 52 putative immunophilins have been identified in *Arabidopsis*, including 29 CYPs, and 23 FKBPs (He et al., 2004). Thus far, the group is the largest family of immunophilin identified in any organism. One feature of *Arabidopsis* immunophilin family that arrests attention is that a large fraction is confined to a particular locality of the chloroplast and that up to 11 FKBPs and 5 CYPs were predicted to exist in the thylakoid lumen. The sequences divergence implies that they may have highly specialized functions in thylakoid lumen (Romano et al., 2005; Edvardsson et al., 2007).

The CYP38 protein is a complex immunophilin residing in the *Arabidopsis* thylakoids lumen. Its ortholog from *Spinacia*, TLP40, is also discovered to be located in the thylakoids lumen (Fulgosi et al., 1998), which is intended to function in the phosporylation of thylakoid proteins (Vener et al., 1999; Rokka et al., 2000). Recently, it is shown that CYP38 takes up a vital role in the assembly and maintenance of PSII supercomplexes in *Arabidopsis* (Fu et al., 2007), and its crystal structure is also found (Vasudevan et al., 2012). CYP38 deficiency is found to be able to severely decrease the *in vivo* phosphorylation of PSII core proteins (Sirpiö et al., 2008). Sirpiö et al. (2008) also suggests that during assembly of PSII, CYP38 protein directs the appropriate folding of D1 (and CP43) into PSII, thereby making the accurate assembly of the water-splitting Mn_4_-Ca cluster able, even with high PSII turnover. It is still a void in many studies, however, when it comes to a mechanistic analysis of plant response to HL stress, especially to how the signaling pathway of CYP38 protects the PSII.

PsbO, referred to as the ‘manganese-stabilizing’ protein, has been proved to bind with high affinity to GTP (Spetea et al., 2004) and function as a GTPase (Lundin et al., 2007a). From cyanobacteria to higher plants this extrinsic subunit is conserved. There are two different protein isoforms, PsbO1 and PsbO2, have been discovered in *Arabidopsis*, and they differ only by 10 amino acids in the mature form. It is found in further study that the PsbO2 component demonstrated considerably higher GTPase activity than PsbO1 protein (Lundin et al., 2008). The different functions of PsbO1 or PsbO2 are understood with the help of two T-DNA insertion mutant lines of *Arabidopsis* deficient in either isoform: it seems that PsbO1 mainly supports PSII activity, whereas the PsbO2 regulates the dephosphorylation and turnover of D1 protein (Lundin et al., 2007b). However, studies on the molecular mechanisms that underlie the roles of PsbO2 in response to abiotic stresses are poor.

It is believed that reactive oxygen species (ROS) and calcium (Ca^2+^) are quite essential signaling messengers in plant cells (Bowler and Fluhr, 2000; Romeis et al., 2001). calmodulin (CaM), a ubiquitous second messenger, serves as a crucial sensor for Ca^2+^ in signal transduction in plants. CaM in *Arabidopsis* varies in different isoforms and they can interact with their particular targets upon different exogenous stimuli (Bowler and Fluhr, 2000; Romeis et al., 2001; Li et al., 2012). However, analysis of the ROS and Ca^2+^-CaM cascade response to HL, especially the signaling pathway, is still lacking.

This paper probes into the possible molecular mechanisms of the protective roles of CYP38 on PSII under the HL stress process. The result of the study showed that *CYP38* gene transcription was up regulated in HL condition. It is also discovered that PsbO2 may a target inhibited by CYP38, and the role of CYP38 in HL-induced defense response is important.

## Materials and Methods

### Plant Materials, Growth Conditions, and Chemicals

Wild-type *Arabidopsis* ecotype Columbia (Col-0) plants, *cyp38* (SALK_029448), *psbo1* (SALK_093396), *psbo2* (SALK_024720) knockout mutants and the CYP38 complementation line (*cyp38-C*), were used in this study. The seeds of *cyp38* and the *cyp38-C* line were obtained from Professor Eva-Mari Aro. and mutant seeds *psbo1, psbo2* were obtained from the European *Arabidopsis* Stock Center. Following 2 d of cold stratification, seeds were grown in an environmentally controlled growth room at 22°C with a 16/8 h day/night cycle and 82% relative humidity. Fluo-3-AM and Singlet Oxygen Sensor Green Reagent (SOSG) were obtained from Molecular Probes (Eugene, OR, USA). BAPTA-AM, cyclopiazonic acid (CPA), ascorbic acid (AsA), 2′7′-dichlorofluorescin diacetate (H_2_DCFDA) and cyclosporin A (CsA), were purchased from Sigma-Aldrich (Shanghai, China).

### High Light Treatment

*Arabidopsis* plants were fully exposed to HL (2000 μmol photons m^-2^ s^-1^) supplied by light-emitting diode panels (Photon System Inst.) (Zhao et al., 2014). Protoplasts at a chlorophyll concentration of 0.2 mg ml^-1^ were illuminated with white light at 2000 μmol photons m^-2^ s^-1^ at 20°C (Herbstová et al., 2012). Control samples of Protoplasts were kept in the dark or normal light, and otherwise identical conditions. Isolated thylakoid membranes (0.1 mg Chl ml^-1^) were illuminated for up to 1 h with 2000 μmol photons m^-2^ s^-1^ at 20°C. The *Arabidopsis* plants used for qRT-PCR were illuminated with 2000 μmol photons m^-2^ s^-1^ at 20°C for up to 15 h.

### DAB Staining

For ROS detection, the leaves from wide type (WT) plants and *cyp38* mutant plants were incubated in DAB solution (1 mg ml^-1^) in the dark for 8 h (Liu et al., 2007). The leaves were then cleared using 95% ethanol for 10 min at 80°C to remove the chlorophyll completely.

### Measurement of Chlorophyll Content and Photochemical Efficiency

Chlorophyll from leaves, Protoplasts or thylakoid membranes was extracted by 95% ethanol at 80°C. The chlorophyll content in each unit mass or unit volume of sample was determined as described by Lichtenthaler (1987), and then counting or concentration adjusting was performed, so that it would be more convenient to release the data or perform the next stage of the experiment. Integrity of PSII affected the ratio of maximum variable fluorescence to maximum yield of fluorescence (Fv/Fm), which was measured by a PAM fluorometer (Walz GmbH, Effeltrich, Germany) (Ralph et al., 2005).

The content was determined spectrophotometrically using the formula Chl (a + b) = 5.24 *A*_664.2_ + 22.24 *A*_648.6_, the content of Chlorophyll (%) = Chl (a + b) ×*V* × 10^-3^/m, where Chl (a + b) equaled the chlorophyll concentration in in μg ml^-1^, *A* represented the absorption, *V* was the volume of the 95% ethanol and *m* symbolized the mass of individual leaves.

### Total RNA Extraction and Quantitative Reverse Transcript-PCR (qRT-PCR)

Following the protocol of the manufacturer of TRIzol reagent (Sigma), total RNA was extracted, at times as indicated subject to different treatments, from detached leaves utilizing the said reagent. The RNA concentration and purity were determined, with OD at 260 nm. Total RNAs were transcribed reversely into cDNAs using an M-MLV kit (Takara), and the cDNAs were adopted as polymerase chain reactions templates. For qRT-PCR assay, SYBR green RT-PCR amplifications were conducted using a total volume of 20 μl that contains 10 μl of SYBR Green real-time PCR Master mix (Toyobo), 5 μl of cDNA, 0.5 μl each of forward and reverse primers (10 μM), and 4 μl of distilled water. The *Actin* (Zhou et al., 2013) was amplified as an endogenous control. PCR amplification was performed with the light cycler 2.0 instrument (Roche Applied Science). The gene-specific primers for RT-PCR analysis were employed as depicted in the Supplementary Table S1. Each measurement of gene expression profiles was carried out at least three times with independent experimental replicates.

### Treatment with CsA

Before HL treatment, the *Arabidopsis* leaves, protoplasts or thylakoid membranes from different samples were pre-sprayed with solutions containing CsA. CsA was used at a final concentration of 10 μM.

### Isolation of Thylakoid Membranes and GTP Hydrolysis Assay

Thylakoid membranes from the WT, *cyp38, psbo1*, and *psbo2* were isolated as previously described (Robinson et al., 1981). To assess the GTP hydrolysis activity of PsbO protein, PSII membranes were isolated from different type plants finally at a same chlorophyll content for each sample as previously described (Berthold et al., 1981; Lundin et al., 2007a). GTP hydrolysis assay using a QuantiChrom^TM^ GTPase Assay Kit (DATG-200, Hongyue, China).

### Western Blot

For Western blotting, the protein samples of thylakoid membranes, at a consisitent chlorophyll content, treated with or without HL (2000 μmol photons m^-2^ s^-1^) were transferred to poly (vinylidene difluoride) membranes (PVDF-Plus; Micron Separations, Westborough, MA, USA), and reacted with specific anti-D1 antibody (Cell Signaling Technology) followed by an enhanced chemiluminescence (ECL-Plus) detection system (Amersham Biosciences, Chalfont St Giles, UK), then analyzed by chemiluminescence imaging (LAS-1000; Fuji, Japan).

### Protoplast Isolation

Protoplast isolation as described previously (He et al., 2007; Li et al., 2012). The purified protoplasts were suspended in the W5 solution (154 mM NaCl, 5 mM KCl, 125 mM CaCl_2_, 5 mM Glc, and 1.5 mM MES-KOH, pH 5.6).

### Laser Confocal Scanning Microscopy (LCSM)

Reactive oxygen species production was determined by detecting the fluorescence of DCF, the product of oxidation of H_2_DCFDA, as described previously (Gao et al., 2008). Ca^2+^ content of the protoplasts was determined by detecting the fluorescence of Fluo-3, the product of Fluo-3-AM (Zhang et al., 1998). With or without HL treatment, the *Arabidopsis* protoplasts were incubated with H_2_DCFDA or Fluo-3-AM at a final concentration of 5 μM. The intracellular ROS production and distribution, as well as the chloroplast autofluorescence and Fluo-3 fluorescence, were visualized under the Zeiss LSM 510. The fluorescence intensity at 525 nm was used to determine the relative DCF and Fluo-3, and chloroplast autofluorescence was visualized at 650 nm with a long pass filter, all this with an excitation wavelength of 488 nm.

### Flow Cytometry Analysis

Singlet oxygen (^1^O_2_) react with the SOSG to produce SOSG endoperoxides (SOSG-EP), as described previously (Shen et al., 2011). To the changes in DCF, Fluo-3, and SOSG-EP, flow cytometry FACScanto II (Becton Dickinson, Mountain View, CA, USA) equipped with BD FACSDiva acquisition soft-ware was used. Protoplasts were incubated with 10 μM of SOSG, 5 μM H_2_DCFDA, or 5 μM Fluo-3-AM for 30 min at room temperature and then analyzed by flow cytometry. The fuorescence of SOSG-EP was excitated with a 504 nm laser. Autofuorescence of the unstained protoplasts under the same physiological conditions were used as controls. Histograms were processed with FCS Express software. Fluorescent effux was measured by counting cells in the H region of the plot (Chen et al., 2006).

## Results

### HL Causes Inhibition of Photosynthesis in *Arabidopsis*

**FIGURE 1:**
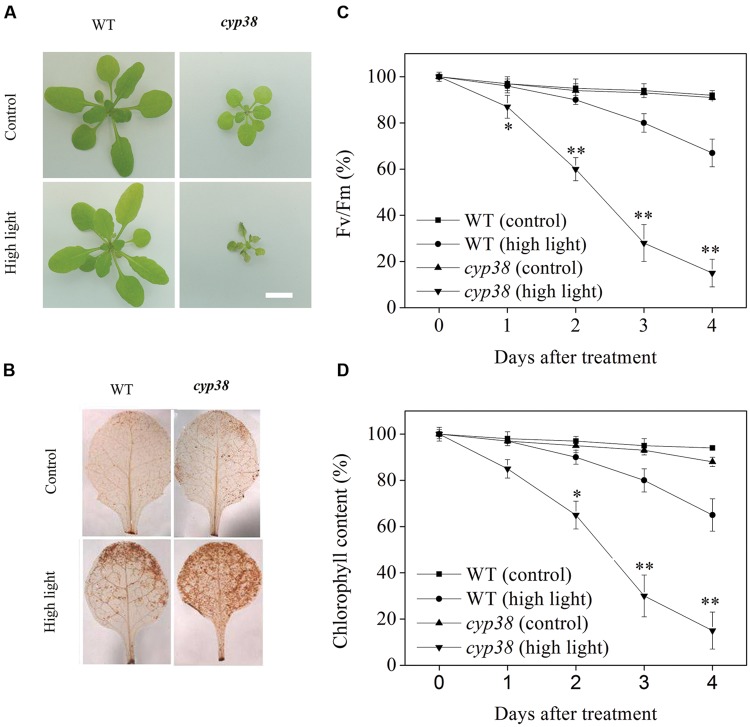
**The *cyp38* mutant shows defect in photosynthesis compared with WT plants. (A)** Phenotype of WT plants and *cyp38* mutant plants with or without high light (HL) treatment (scale bar = 1 cm). **(B)** DAB staining of *Arabidopsis* leaves from **(A)**. **(C,D)** Photochemical efficiency and chlorophyll content of detached leaves from WT and *cyp38* mutant with or without HL treatment at indicated times. Fv/Fm, maximum quantum yield of PSII electron transport (maximum variable fluorescence/maximum yield of fluorescence). Asterisks (^∗^) indicate a significant difference from the Control at *^∗^P* < 0.05, ^∗∗^*P* < 0.01. Error bars are ±*SD* values for three replicates.

It is reported in literatures that deletion of CYP38 causes stunted growth of the plants and the *cyp38* mutant plants is highly sensitive to HL (Fu et al., 2007; Sirpiö et al., 2008). When *Arabidopsis* plants were treated under HL condition, as shown in the **Figure 1A**, *cyp38* mutant plants soon wilt and finally die. However, when the *CYP38* gene was restored, the wilting and death of the plants (*cyp38-C* plants) were eased off (data not shown). Further DAB staining showed that, under HL condition, as the *cyp38* mutant was dying, its leaves accumulated more ROS in comparison with WT (**Figure 1B**). Stunted growth, as well as high sensitivity to HL of the *cyp38* mutant, illustrate that, because of CYP38 deletion, the photosynthetic activity of plants is more likely to be damaged by oxidation induced by HL. For further data analysis, we have simultaneously detected the chlorophyll content of leaves and photochemistry efficiency of PSII. As shown in the **Figures 1C,D**, under HL treatment, there was dramatic decline in chlorophyll content and photochemistry efficiency of the *cyp38* mutant. After HL treatment for 3 days, the chlorophyll contents in WT plants and *cyp38* mutant plants, respectively, fell down to 80 and 28% of the original levels, and the photochemistry efficiencies, respectively, fell down to 83 and 30% of the original levels.

### CYP38 is Involved in Response to HL Stress Condition in *Arabidopsis*

**FIGURE 2:**
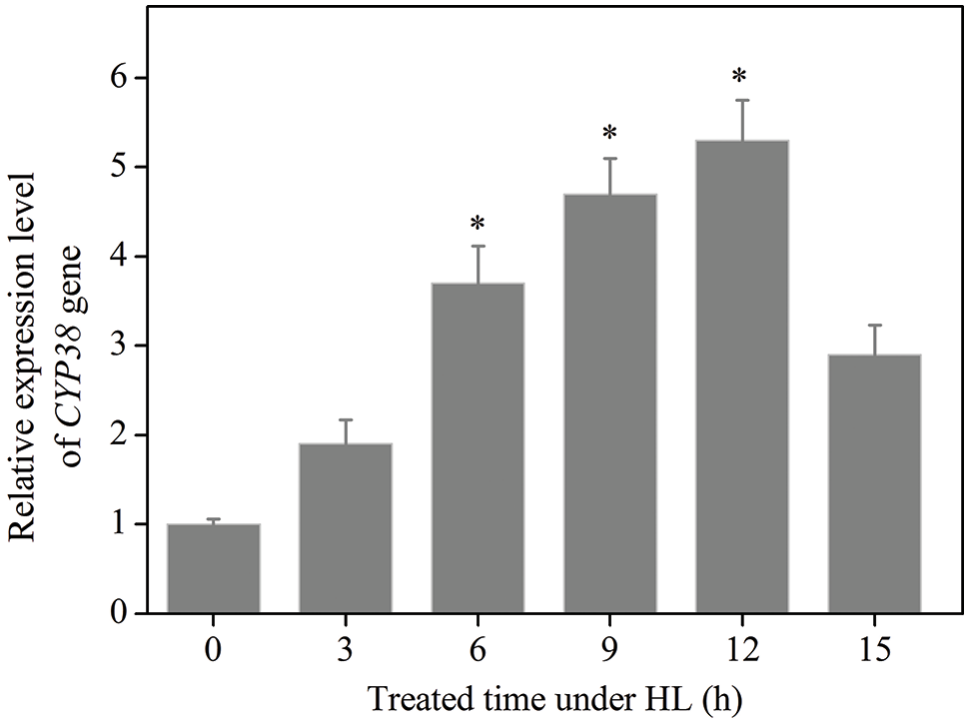
**Changes in *CYP38* gene expression in HL treated WT *Arabidopsis* plants.** qRT-PCR analysis of the expression of *CYP38* gene during 15 h in the HL-treated WT plants. After HL treatment, total RNAs were extracted from the HL-treated plants at indicated times. Error bars are ±*SD* values for three replicates. Asterisks (^∗^) indicate a significant difference from the control at ^∗^*P* < 0.05.

The data given above illustrates that, in terms of protecting photochemistry efficiency and HL resistance of plant, CYP38 plays an important role. In order to further confirm with experiment that CYP38 essentially participates in the protection of photosynthetic activity under HL, we detected the change of *CYP38* gene expression level in WT plant after HL treatment. qRT-PCR results showed that, after HL treatment, *CYP38* gene expression arose, and rose to a level four times higher than that in the control group at 6 h (**Figure 2**). To verify the authenticity of Result of RT-PCR, we added *Rbcl* gene (Kress and Erickson, 2007) and *UBQ* gene (Miki et al., 2005), respectively, as control groups (Supplementary Figure S4), and found that *CYP38* gene expression levels are consistent with the *Actin* group. On the contrary, the gene expression under low light (LL) is not significantly different from that of control genes (Supplementary Figure S1). In sum, CYP38 may, as an important regulation molecule, participate in the protection of *Arabidopsis* plant under HL condition.

### Lack of CYP38 Gene Increases HL-Induced Chloroplast ROS Production

**FIGURE 3:**
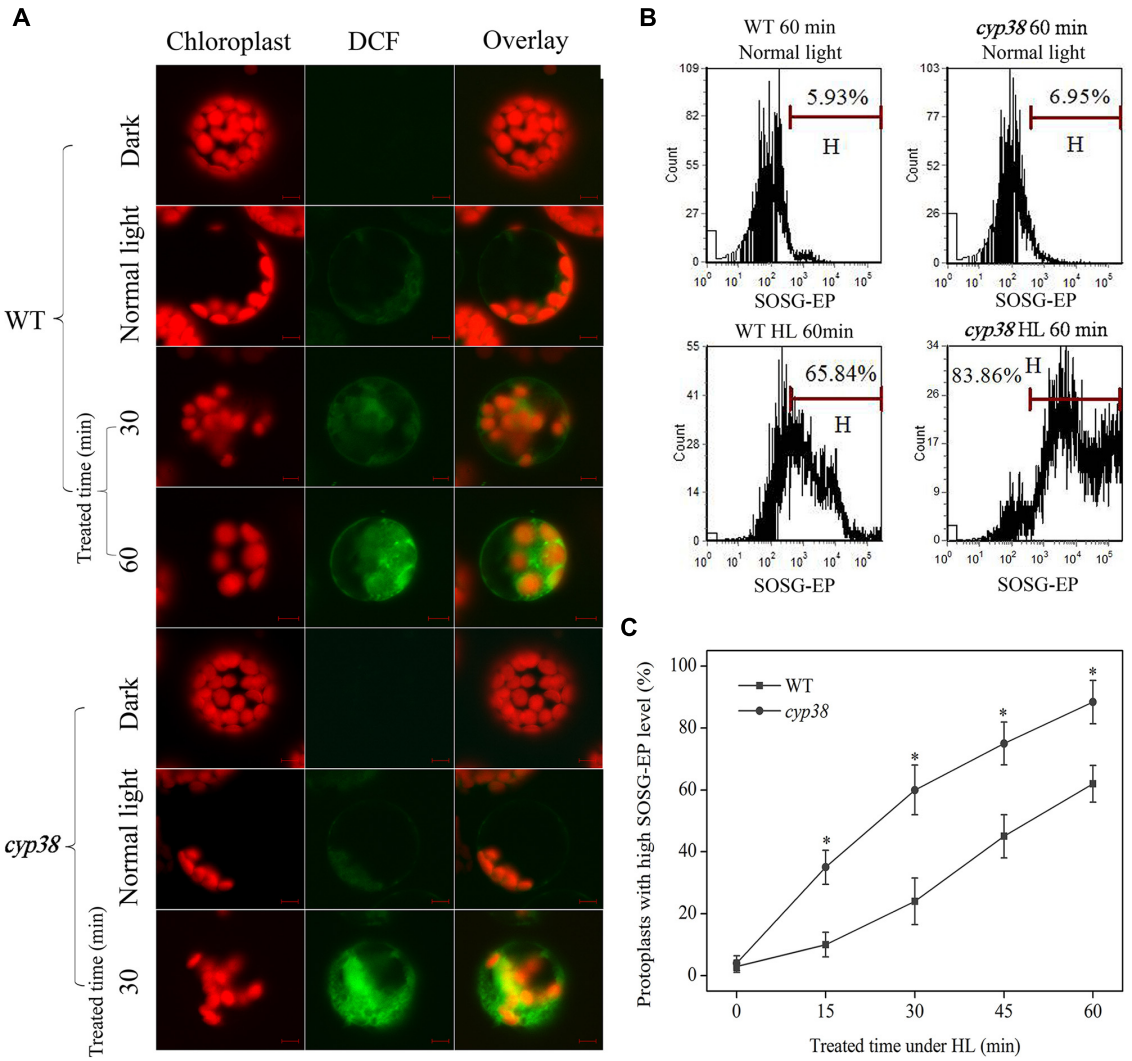
**Changes in ROS level under HL treatment. (A)** Sub-cellular localization of HL-induced ROS production. Protoplasts from different plants were treated with or without HL for the indicated time period, then incubated with H_2_DCFDA (at a final concentration of 5 μM) for 30 min at room temperature, and observed using LCSM as described in materials and methods. Protoplasts derived from WT and *cyp38* mutant without HL treated were kept in dark and normal light as controls. Other protoplasts were treated with HL (2000 μmol photons m^-2^ s^-1^) up to 60 min. Chloroplast autofluorescence is false colored red, and DCF fluorescence is false colored green. Scale bars = 10 μm. **(B)** Analysis of ^1^O_2_ in HL-treated WT and *cyp38* protoplasts by flow cytometry using fuorescence probe SOSG. The marker bar H was set to indicate the cells with high fuorescence effux. SOSG-EP effux was measured by counting cells in the H region of the plot. **(C)** Kinetics of changes in ^1^O_2_ levels by flow cytometry analysis in WT and *cyp38* protoplasts under HL treatment. Asterisks indicate a signifcant difference from the control by *t*-test: ^∗^*P* < 0.05.

Under HL condition, the process of damage to photosynthetic activity of *cyp38* mutant plants, as well as death of the plants later on, was characterized by outbreak of excessive ROS in leaves (**Figure 1B**). These illustrate that CYP38 plays a positive role in maintaining the redox state of the leaves. Cells are the most fundamental unit for plants’ function. In order to carry out deeper research on relevant mechanisms, it is necessary to do ROS distribution analysis at cellular or even sub-cellular level. The imaging results showed that, after 30 min of HL treatment, there was generation of ROS in protoplasts derived from WT plants; after 60 min, significance in quantity was observed (**Figure 3A**). The inhibition of photosynthetic chain can induce ^1^O_2_ to synthesize. To clarify the type of ROS, it is found after flow cytometry analysis that, lots of ^1^O_2_ was synthesized in the cell during the early stage of HL processing (**Figures 3B,C**). As ^1^O_2_ is of highly oxidation activity, high cell synthesis is the cause of overdue impairment of cells. Under HL condition, the cell generated ROS, indicating that HL causes change of the redox state of cells. In comparison to this, a higher outbreak of protoplast ROS derived from *cyp38* mutant plants occurred as early as 30 min after HL treatment, while DCF fluorescence imaging and chloroplasts were subject to co-localization (**Figure 3A**). Furthermore, CsA, as the non-selective combinator and inhibitor of CYPs (Handschumacher et al., 1984; Steinmann et al., 1991), can be used for pre-treatment of protoplasts derived from WT plants (Supplementary Figure S2). It was discovered that, after 30 min of illumination (HL) in the pre-treatment, the ROS which was larger than that non-pretreated group. The outbreak of ROS which is subject to co-localization with chloroplasts in CYP38-deletant under HL may be an important reason for lack of photosynthetic efficiency and poor HL resistibility in *cyp38* mutant plant.

### D1 Protein is Drastically Decreased in CYP38-Deletion Plants Under HL Stress Condition

**FIGURE 4:**
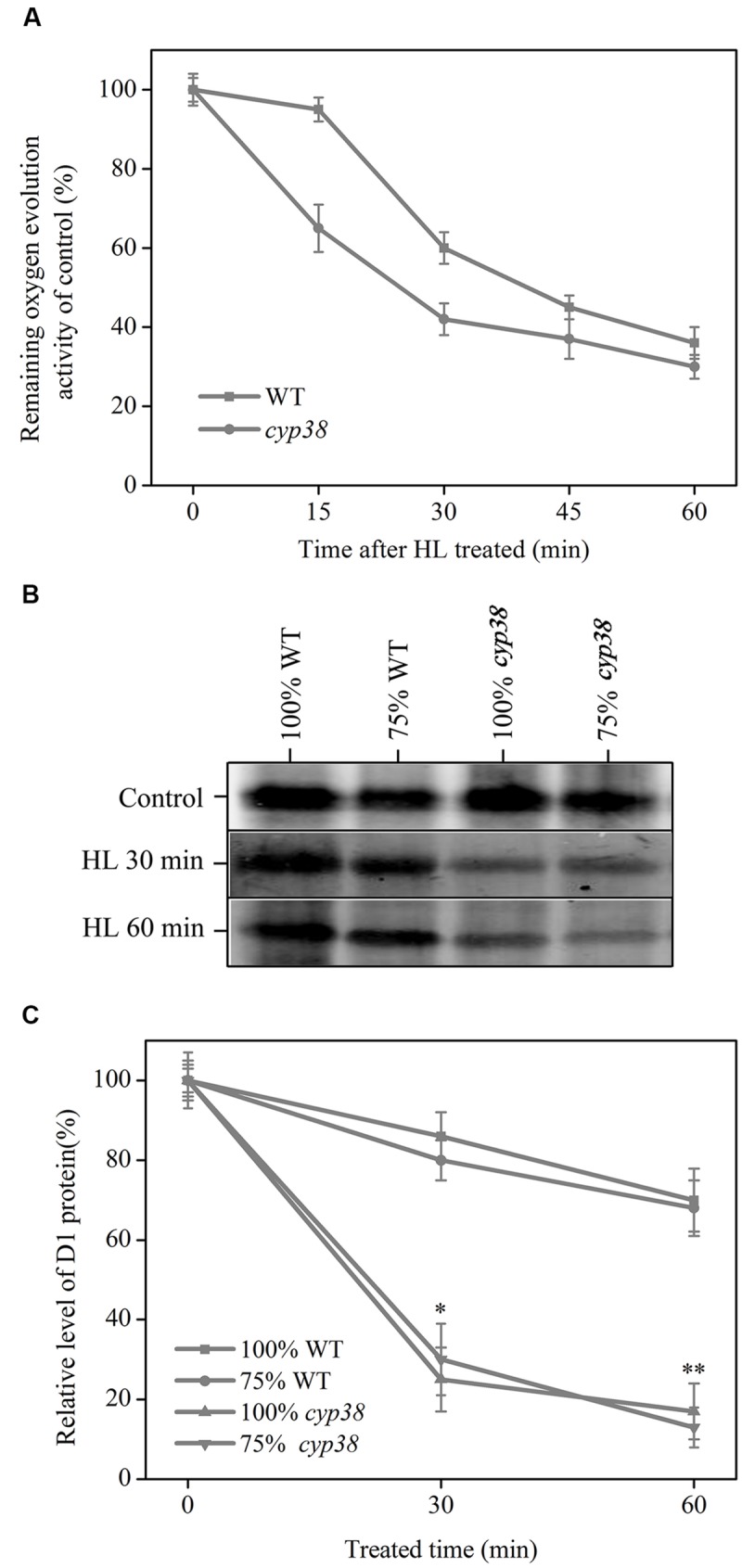
**Kinetics for HL-induced inactivation of PSII oxygen evolution and D1 protein degradation in WT and *cyp38* mutant. (A)** Relative levels of oxygen evolution activity in isolated thylakoid membranes of WT and *cyp38* mutant. **(B)** Immunoblot of the D1 protein isolated from WT and *cyp38* plants. Thylakoid membranes sample content 0.2 μg chlorophyll was set to 100%. **(C)** Quantitative analysis of the result in **(B)**. The standard deviations were calculated for data obtained from three independent experiments. Asterisks (^∗^) indicate a significant difference from the Control at ^∗^*P* < 0.05, ^∗∗^*P* < 0.01.

Oxygen-evolving complex (OEC), as an important constituent of PSII, can become a major damaged point (Hakala et al., 2005), whether under biotic or abiotic stress, leading to the decrease in activity of oxygen evolution. Studies also proved that, D1, as one of the core proteins of PSII, boasts the fastest turnover rate and is the part which is most likely to be damaged by oxidation (Greenberg et al., 1989; Jegerschoeld et al., 1990). Our research proved that CYP38 may plays a certain role in maintaining the redox state of chloroplast, and deletion of this protein induce excessive generation of chloroplast ROS (**Figure 3**). In order to offer more in-depth information, the thylakoid membranes of WT and *cyp38* mutant were extracted in this study, and treated under HL up to 60 min, in order to detect the oxygen evolution activity and remaining D1 protein content. As shown in the **Figure 4A**, under HL treatment, the activity of oxygen evolution of *cyp38* mutant fell distinctly. Instead, the WT showed a lag phase of 15 min, and reached similar level as in *cyp38* mutant after 45 min of HL treatment. Both the D1 contents of WT and *cyp38* mutant declined, yet there was distinctive difference between them. In 60 min, the inventories of D1 protein in the extracted membranes of WT and *cyp38* mutant, respectively, fell down to 70 and 15% of the original levels (**Figures 4B,C**). The detection of the level of remaining protein after HL treatment showed that D1 protein degraded rapidly in *cyp38* mutant. This may be caused by over-oxidation of chloroplasts caused by CYP38 deletion. The mechanism behind the more significant reduction in D1 proteins in *cyp38* mutant as compared with WT was further investigated.

### HL-Induced PsbO2 Transcription and Activation Can be Enhanced by CsA

**FIGURE 5:**
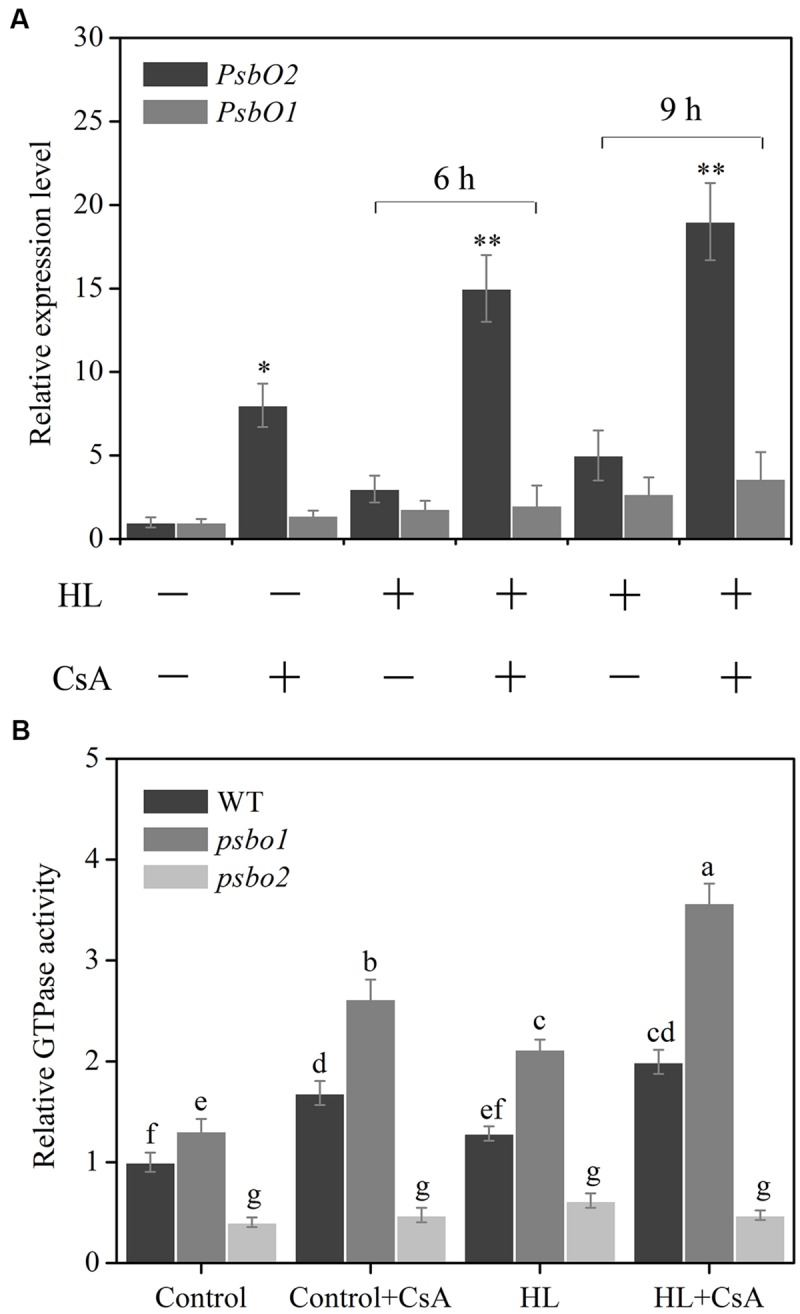
**Effect of CsA on HL-induced *PsbO* gene expression and its GTPase activation in *Arabidopsis*. (A)** qRT-PCR analysis of *PsbO1* and *PsbO2* gene expression in CsA-pretreated detached leaves of WT plants after HL treatment. Total RNAs were extracted from the HL-treated detached leaves at the indicated time for the qRT-PCR analysis. **(B)** GTPase activity analysis using CsA-pretreated PSII membranes of WT, *psbo1* and *psbo2* after HL treatment. Detached *Arabidopsis* thaliana leaves or PSII membranes were pre-incubated with or without CsA (10 μM), and then treated with HL. WT GTPase activity at control light was set to 1. Different letters indicate statistically signifcant differences between treatments (Duncan’s multiple range test: *P* < 0.05). Asterisks (^∗^) indicate a significant difference from the Control at *^∗^P* < 0.05, ^∗∗^*P* < 0.01.

D1 dephosphorylation is a step of the degradation damaged D1, and degradation is impossible if dephosphorylation is abnormal (Rintamäki et al., 1996). PsbO2, as a membrane protein of PSII, is proved to can positively regulate D1 dephosphorylation in recent research report (Lundin et al., 2007b). Our data given above indicates that CYP38 participates in the turnover regulation of D1 (**Figure 4**). In addition, the results of Sirpiö et al. (2008) indicate that CYP38 imposes regulation effect on D1 phosphorylation. In order to elucidate if there is an interrelation between CYP38 and PsbO2, CsA, the common combinator and inhibitor of CYPs (Handschumacher et al., 1984; Steinmann et al., 1991), was used. First, the change in transcript level of PsbO2 was detected using qRT-PCR. Results showed a significant increase in PsbO2 expression in detached *Arabidopsis* leaves pretreated with CsA at the 6 h and 9 h under HL treatment, compared to that of the non-pretreated samples (**Figure 5A**). While the transcription of *PsbO1* has no much change under HL or CsA pretreated conditions. The change of PsbO activity under HL was analyzed using two T-DNA insertion mutant lines, *psbo1* and *psbo2*. In CsA-pretreated PSII membranes, the distinctive increase of PsbO activity was found at the 6 h after HL treatment in *psbo1* mutant compared to that WT and *psbo2* mutant (**Figure 5B**). These data indicates that CYPs cascades are involved in HL-induced PsbO activation of and transcription, and PsbO2 may be the main target.

### CYP38 is Required for Inhibiting HL-Induced PsbO2 Activity

**FIGURE 6:**
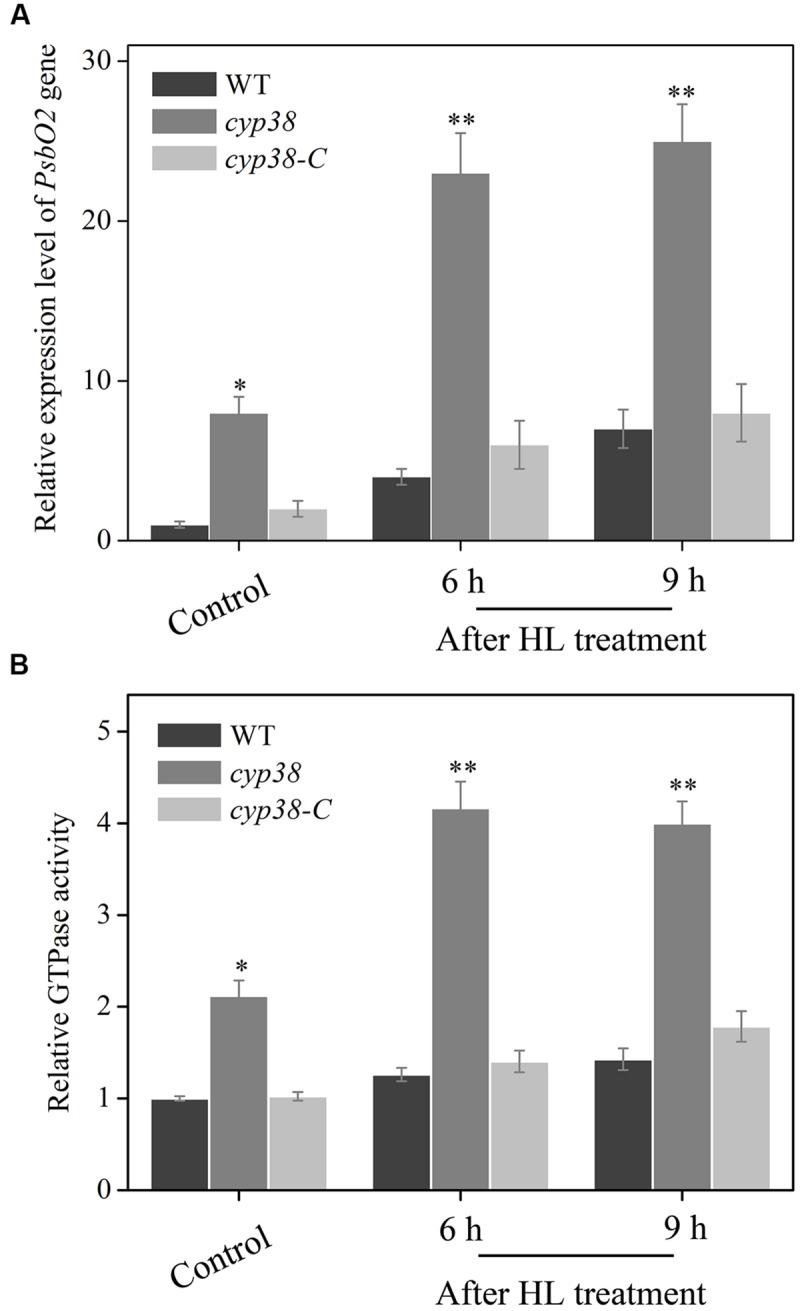
**Effect of CYP38 on HL-induced *PsbO2* gene expression and its GTPase activation in *Arabidopsis*. (A)** qRT-PCR analysis of *PsbO2* gene expression in plants of WT, *cyp38* and *cyp38-C* with or without HL treatment. **(B)** GTPase activity analysis using PSII membranes extracted from WT, *cyp38* and *cyp38-C*, then treated with or without HL for subsequent test. WT GTPase activity at control light was set to 1. Error bars are ± *SD* values for three replicates. Asterisks (^∗^) indicate a significant difference from the control at *^∗^P* < 0.05, ^∗∗^*P* < 0.01.

Data given above illustrates that CYPs may participate in regulation of HL-induced gene expression and activity of PsbO2 (**Figure 5**). However, further clarification is needed in order to confirm whether CYP38 participates in the process. In order to explain whether CYP38 involves regulation activity of PsbO2, *cyp38* mutant and *cyp38-C* were utilized simultaneously in the experiment. First of all, under HL condition, we detected *PsbO2* gene expressions in *cyp38* mutant and *cyp38-C* (**Figure 6A**). The results showed that *PsbO2* gene expression in *cyp38* mutant was higher than that of WT and *cyp38-C*, and such distinction became even larger after HL treatment. However, contrary to this, there was no distinctive change of *PsbO1* gene expression was observed either in *cyp38* mutant or *cyp38-C* (Supplementary Figure S3). The detection and analysis of PsbO activity was carried out at the same time. In *cyp38* mutant, PsbO activity rose distinctly after HL treatment (**Figure 6B**). In *cyp38-C*, PsbO activity was similar to that of WT. In sum, CYP38 has an inhibiting effect on PsbO activity and influence of its transcription, the main target is PsbO2.

### Gene Expression of CYP38 is Related to Ca^2+^-CaM3

**FIGURE 7:**
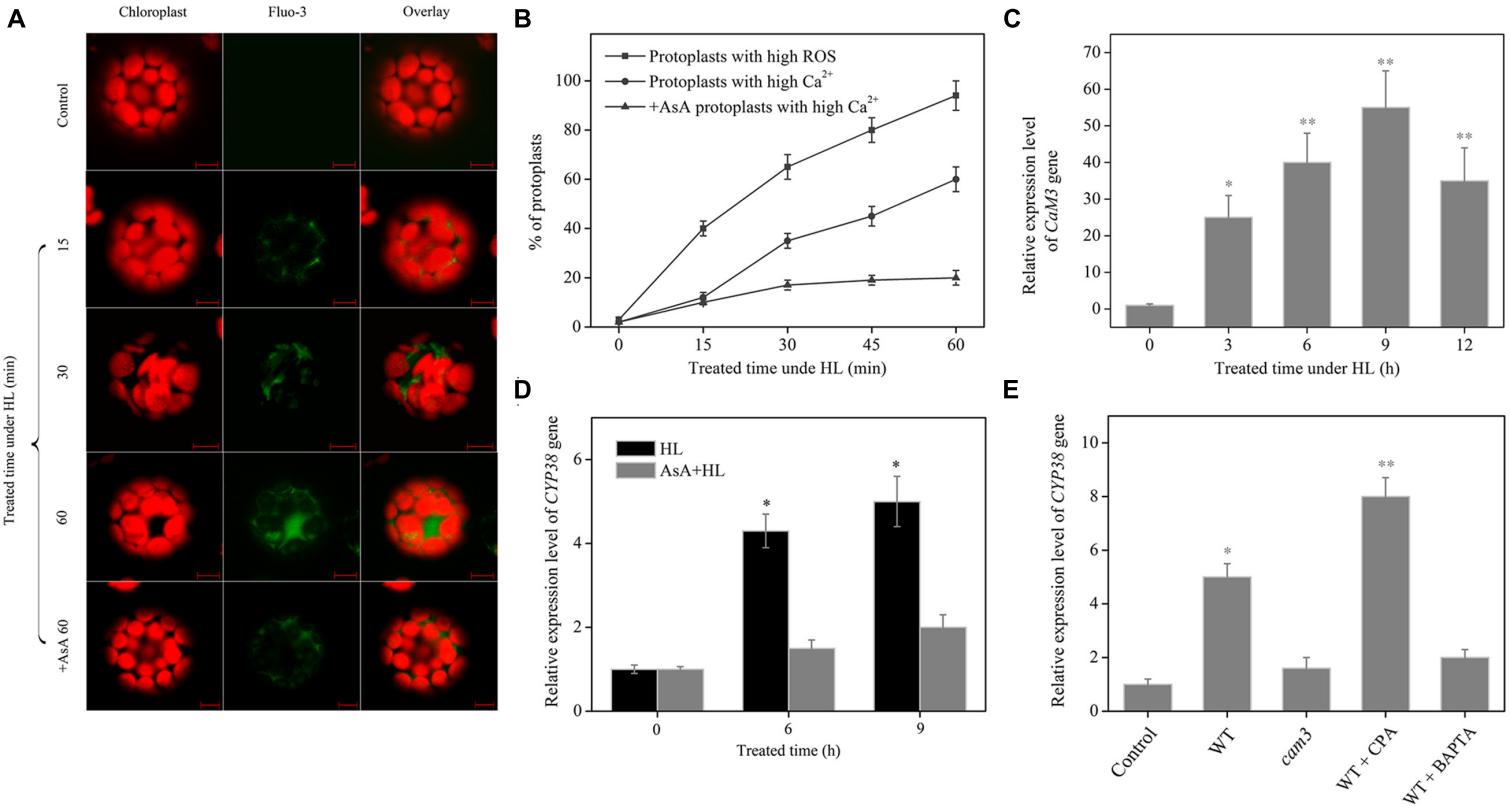
**Effect of ROS and Ca^2+^-CaM3 cascade on *CYP38* gene expression. (A)** Sub-cellular localization and content of HL-induced Ca^2+^ production. Protoplasts were treated with or without HL for the indicated time period, then incubated with Fluo-3-AM for 30 min at room temperature, and observed using LCSM as described in materials and methods. Chloroplast autofluorescence is false colored red, and Fluo-3 fluorescence is false colored green. Scale bars = 10 μm. **(B)** Kinetics of changes in ROS and Ca^2+^ level by flow cytometry analysis after HL treatment. **(C)** Changes in *CAM3* gene expression in HL-treated *Arabidopsis* WT plants. **(D)** *CYP38* gene expression in the detached leaves of WT with or without AsA. **(E)** *CYP38* gene expression in the detached leaves of WT with or without BAPTA-AM (1 mM) or CPA (1 μM) in response to HL treatment. Total RNAs were extracted from the HL-treated plants at the indicated time for the qRT-PCR analysis. Before HL treatment, the protoplasts and detached leaves were incubated with AsA at 1 mM final concentration. values for three replicates and asterisks (^∗^) indicate a significant difference from the Control at *^∗^P* < 0.05, ^∗∗^*P* < 0.01.

Reactive oxygen species, as a widely studied oxidative stress molecular, can also function as cellular second messenger. Recently, it is proved that ROS plays an important role in cell signal transduction, one target being Ca^2+^ (Mori and Schroeder, 2004; Bose et al., 2014). The results showed that, there was a significant increase in the level of ROS in the plant cells under HL conditions, subsequent to the cytoplasmic calcium [Ca^2+^]_cyt_, content increased gradually; after the removal of ROS by AsA, the level of Ca^2+^ significantly dropped (**Figures 7A,B**). These results indicated that ROS may be an important signal molecular to increase the concentration of the [Ca^2+^]_cyt_.

Ca^2+^, as an important second messenger in plant cells, can function under abiotic stresses (Bowler and Fluhr, 2000; Li et al., 2012). We detected an increase in [Ca^2+^]_cyt_ and up-regulation of the *CAM3* gene transcript level under HL condition (**Figures 7A,C**). In order to verify whether this change associated with the function of CYP38, the AsA, BAPTA-AM (Yue et al., 2012), CAP and *cam3* mutant was used. Under HL condition, either scavenging [Ca^2+^]_cyt_ with BAPTA-AM or abolishing ROS by treatment with AsA dramatically declined *CYP38* gene expression (**Figures 7D,E**). The *CYP38* gene expression was also droped in *cam3* mutant plants. On the contrary, rapid [Ca^2+^]_cyt_ elevation was induced by CPA can increase its transcription. The results above indicate that gene expression of CYP38 is related to cell redox signal induced [Ca^2+^]_cyt_-CaM3 cascade.

## Discussion

This study is an attempt to understand the possible molecular mechanisms in order to explain how CYP38 plays an important role in protection of PSII in *Arabidopsis* under HL treatment. The results shown in this paper provide evidence for the cellular signaling cascade of the function of CYP38 in *Arabidopsis* in response to HL stress.

Recent studies have reported that deletion of CYP38 in *Arabidopsis* results in stunted growth and CYP38-deletant is highly sensitive to HL environment (Fu et al., 2007; Sirpiö et al., 2008; **Figure 1A**). The present study observed that, under HL condition, *cyp38* leaves were more likely to be damaged by oxidation than WT leaves (**Figure 1B**). At the same time, experiment at the sub-cellular level showed that, in the protoplasts derived from *cyp38* mutant, there was excessive outbreak of ROS which mainly subject to co-localization with chloroplasts (**Figure 3**). Further detected the change of *CYP38* gene expression under HL, and discovered that its gene expression was increasing under HL condition compared with controlled and low light condition (**Figure 2**; Supplementary Figure S1), indicating that CYP38 plays an important role in the protection of the chloroplasts in HL response.

Sirpiö et al. (2008) have demonstrated that CYP38 deletion drastically decreases the *in vivo* phosphorylation of D1 protein in *Arabidopsis*. This research discovered that, under HL treatment, the degradation rate of D1 protein in *cyp38* mutant was distinctly higher than that of WT (**Figure 4**). Literature reports in the past pointed out that, first of all, the damaged D1 undergos phosphorylation (Koivuniemi et al., 1995; Rintamäki et al., 1995a). As a signal for location change, the D1 undergoing phosphorylation is guided and migrated from grana membranes to stroma lamellae, where D1 undergos dephosphorylation and later on be degraded (Tikkanen et al., 2008). To sum up, CYP38 deletion causes the inhibiting effect on thylakoid membrane phosphatase to disappear, and correspondingly facilitate dephosphorylation of p-D1, and therefore strengthen degradation of the damaged D1. PsbO2 also has been showed to have positive regulation effect on dephosphorylation and turnover of the PSII reaction center D1 protein (Lundin et al., 2007b). CYP38 and PsbO2 play important roles in D1 turnover process, which indicates that there may be some relevance between them (see the discussion below).

CYP38 protein is identified to be present in the thylakoid lumen. However, the research of Sirpiö et al. (2008) show that considerable amount of CYP38 protein is also associated with PSII monomer. As far as we know, these associated proteins of CYP38 with PSII monomer may participate in relevant regulation of membrane protein, rather than merely being a structural composition. Our data presented that *PsbO2* gene expression and its GTPase activation were increased by CsA pro-treatment in WT plants compared with control (**Figure 5**). Further detection in *cyp38* mutant discovered similar results. However, in CYP38 complementation line *PsbO2* gene expression and its GTPase activation fell down (**Figure 6**). We speculate that, the change of *PsbO2* transcription mainly because of the redox diversification of the plant cell related to CYP38. All these indicated that CYP38 may, through inhibition of PsbO2 activity and then influence the dephosphorylation rate of the damaged D1.

Previous studies discovered that, under HL condition, the degradation rate of p-D1 is lower than that of D1 (Rintamäki et al., 1995b). Therefore, as a mode of existence, p-D1 may be more stable than D1. We consider that the high gene expression of CYP38 under HL and inhibition of PsbO2 prevent dephosphorylation-based degradation of p-D1 protein, and as a result they transform D1 to p-D1 which is relatively more stable. In this way, the excessive degradation loss caused by HL is avoided. Other reports note that PSII existing in the form of p-D1 may no longer have the activity for transformation of light energy, however, the energy dissipation of such PSII can be enhanced (Critchley and Russell, 1994), which is beneficial for the adaptation to HL stress. From another perspective, CYP38 expression drops down to certain levels under LL conditions compared with HL (**Figure 2**; Supplementary Figure S1), which may release inhibition of PsbO2 and therefore enhance turnover of the damaged D1, facilitate PSII repair and increase efficiency of photosynthetic activity.

When plants are under abiotic threatening circumstances (such as excessive illumination), concentration of chloroplsts ROS grows and Ca^2+^ levels in the cytosol increases as well (Baxter et al., 2014). If the balance between light absorption and the use of light energy is disturbed, photosynthesis will be further inhibited (Asada, 2006). In addition, the lack of light energy consumption leads to photoinhibition of PSII and enhances generation of ^1^O_2_, which results in sequential reduction to superoxide anion radical ($O_{2}^{\cdot }$-), hydrogen peroxide (H_2_O_2_) and hydroxyl radicals (^∙^OH; Foyer and Noctor, 2000). This study shows that a large amount of ^1^O_2_ is generated in the cells under excessive light. DAB-stained leaves also tell that a great amount of H_2_O_2_ is produced under the same condition (**Figures 1** and **3**). Recently, some articles claim that H_2_O_2_ ‘signatures’, including antioxidant defense signal and well-known cytosolic calcium ‘signatures’ (Bose et al., 2014; Ozgur et al., 2014; Pottosin et al., 2014), may operate in plant signaling networks.

Ca^2+^, as an important messenger in plant cells (Hashimoto and Kudla, 2011), can function under various stimuli (Bowler and Fluhr, 2000; Romeis et al., 2001; Li et al., 2012). Under HL stress, we found that concentration of [Ca^2+^]_cyt_ and *CAM3* gene expression increased, and inhibited [Ca^2+^]_cyt_ by BAPTA-AM or deficient CaM3, which drastically decreased *CYP38* gene expression in *Arabidopsis* under HL condition, however, pre-incubated with CPA, which induced rapid elevation of [Ca^2+^]_cyt_, and drastically increased *CYP38* gene expression. Additionally, the level of [Ca^2+^]_cyt_ and *CYP38* gene expression were significantly inhibited after removal of ROS by AsA, indicating that ROS generation may be a upstream signaling in these process (**Figure 7**). Our data present a cellular signaling cascade, composed of ROS production, [Ca^2+^]_cyt_ increase and the upward regulation of CaM3 transcript level, which function in the upstream of *CYP38* gene expression in response to HL.

## Conclusion

**FIGURE 8:**
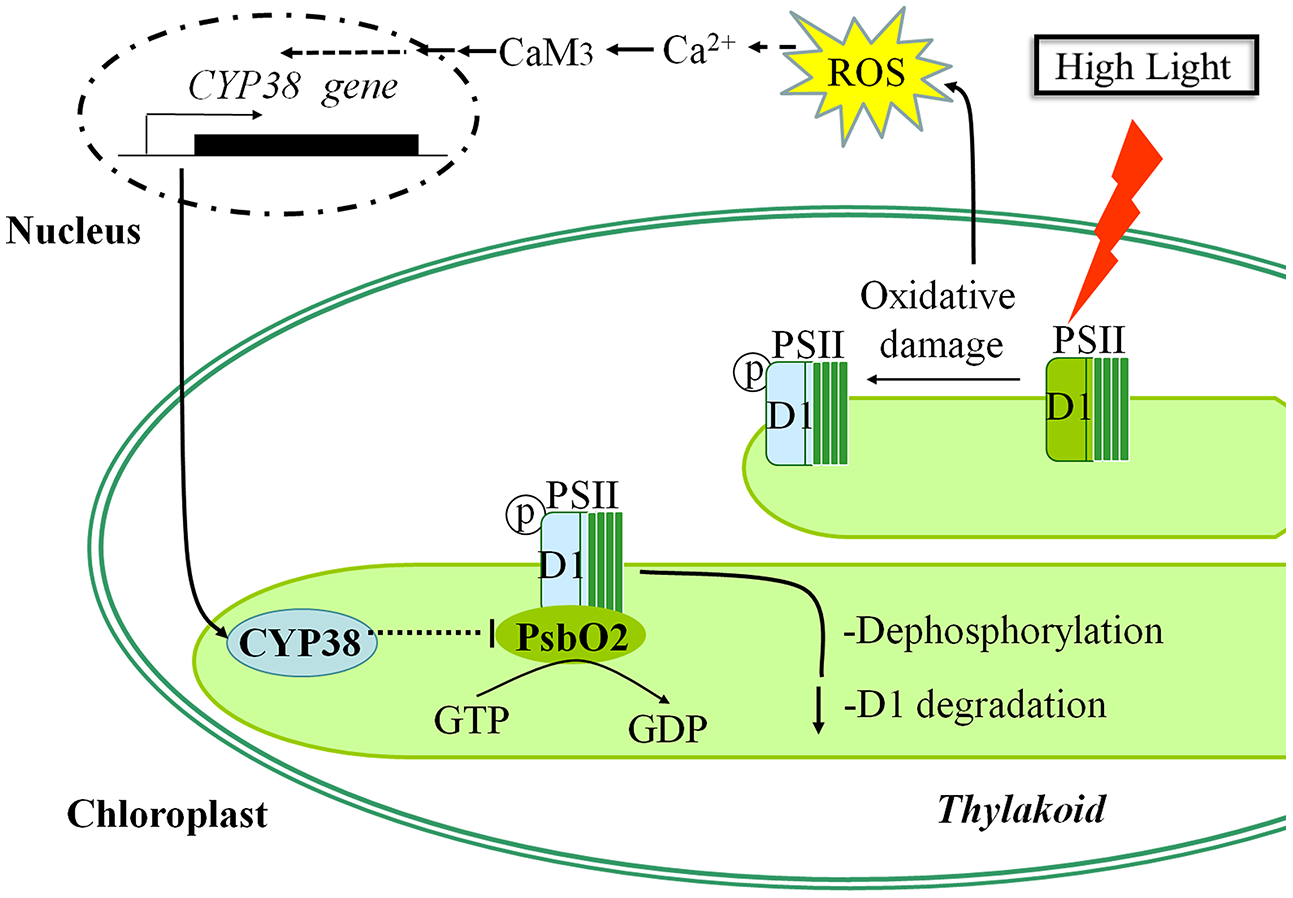
**Model showing the possible signaling pathway for CYP38-induced protection of photosystem II in *Arabidopsis* under high light stress condition**.

Our data showed that CYP38-mediated inhibition of PsbO2 activity modulated the tolerance of *Arabidopsis* to HL stress. According to our experimental results, a potential cascade of cellular events under HL stress was given (**Figure 8**): Firstly, HL stress induced ROS production in chloroplast, causing change to chloroplastic redox state. This signal might cause the increase of [Ca^2+^]_cyt_ and the activation of Ca^2+^-CaM3 cascade, and subsequently facilitated CYP38 expression. The increase in CYP38 greatly inhibited PsbO2 activity, which weakened the dephosphorylation of D1 protein and kept it in a relatively stable phosphorylation state to avoid degradation. These results present a possible mechanism for plants to respond to external HL stress, and shed a new insight into the cellular signaling cascade of CYP38.

## Conflict of Interest Statement

The authors declare that the research was conducted in the absence of any commercial or financial relationships that could be construed as a potential conflict of interest.

## References

[B1] Adir N. Shochat S. Inoue Y. Ohad I. (1990). Mechanism of the light dependent turnover of the D1 protein. *Curr. Res. Photosynth.* 6 1369–1373. 10.1007/978-94-009-0511-5_316

[B2] Aro E. M. Virgin I. Andersson B. (1993). Photoinhibition of photosystem II. Inactivation, protein damage and turnover. *Biochim. Biophys. Acta Bioenerget.* 1143 113–134. 10.1016/0005-2728(93)90134-2 8318516

[B3] Asada K. (2006). Production and scavenging of reactive oxygen species in chloroplasts and their functions. *Plant Physiol.* 141 391–396. 10.1104/pp.106.082040 16760493 PMC1475469

[B4] Barbato R. Friso G. Rigoni F. Dalla Vecchia F. Giacometti G. M. (1992). Structural changes and lateral redistribution of photosystem II during donor side photoinhibition of thylakoids. *J. Cell Biol.* 119 325–335. 10.1083/jcb.119.2.325 1400577 PMC2289643

[B5] Barber J. (2006). Photosystem II: an enzyme of global significance. *Biochem. Soc. Trans.* 34 619–632. 10.1042/BST0340619 17052167

[B6] Baxter A. Mittler R. Suzuki N. (2014). ROS as key players in plant stress signalling. *J. Exp. Bot.* 65 1229–1240. 10.1093/jxb/ert375 24253197

[B7] Berthold D. A. Babcock G. T. Yocum C. F. (1981). A highly resolved, oxygen-evolving photosystem II preparation from spinach thylakoid membranes: EPR and electron-transport properties. *FEBS Lett.* 134 231–234. 10.1016/0014-5793(81)80608-4

[B8] Bose J. Rodrigo-Moreno A. Shabala S. (2014). ROS homeostasis in halophytes in the context of salinity stress tolerance. *J. Exp. Bot.* 65 1241–1257. 10.1093/jxb/ert430 24368505

[B9] Bowler C. Fluhr R. (2000). The role of calcium and activated oxygens as signals for controlling cross-tolerance. *Trends Plant Sci.* 5 241–246. 10.1016/S1360-1385(00)01628-9 10838614

[B10] Chen X. P. Wang Q. Guan J. Huang Z. Y. Zhang W. G. Zhang B. X. (2006). Reversing multidrug resistance by RNA interference through the suppression of MDR1 gene in human hepatoma cells. *World J. Gastroenterol.* 12 3332–3337. 16733848 10.3748/wjg.v12.i21.3332 PMC4087887

[B11] Critchley C. Russell A. W. (1994). Photoinhibition of photosynthesis in vivo: the role of protein turnover in photosystem II. *Physiol. Plant.* 92 188–196. 10.1111/j.1399-3054.1994.tb06670.x

[B12] Edvardsson A. Shapiguzov A. Petersson U. A. Schröder W. P. Vener A. V. (2007). Immunophilin AtFKBP13 sustains all peptidyl-prolyl isomerase activity in the thylakoid lumen from *Arabidopsis thaliana* deficient in AtCYP20-2. *Biochemistry* 46 9432–9442. 10.1021/bi700426q 17655280

[B13] Foyer C. H. Noctor G. (2000). Tansley Review No. 112. Oxygen processing in photosynthesis: regulation and signalling. *New Phytol.* 146 359–388. 10.1046/j.1469-8137.2000.00667.x

[B14] Fu A. He Z. Cho H. S. Lima A. Buchanan B. B. Luan S. (2007). A chloroplast cyclophilin functions in the assembly and maintenance of photosystem II in *Arabidopsis thaliana*. *Proc. Natl. Acad. Sci. U.S.A.* 104 15947–15952. 10.1073/pnas.0707851104 17909185 PMC2000425

[B15] Fulgosi H. Vener A. V. Altschmied L. Herrmann R. G. Andersson B. (1998). A novel multi-functional chloroplast protein: identification of a 40 kDa immunophilin-like protein located in the thylakoid lumen. *EMBO J.* 17 1577–1587. 10.1093/emboj/17.6.1577 9501079 PMC1170505

[B16] Gao C. Xing D. Li L. Zhang L. (2008). Implication of reactive oxygen species and mitochondrial dysfunction in the early stages of plant programmed cell death induced by ultraviolet-C overexposure. *Planta* 227 755–767. 10.1007/s00425-007-0654-4 17972096

[B17] Greenberg B. M. Gaba V. Canaani O. (1989). Separate photosensitizers mediate degradation of the 32-kDa photosystem II reaction center protein in the visible and UV spectral regions. *Proc. Natl. Acad. Sci. U.S.A.* 86 6617–6620. 2671998 10.1073/pnas.86.17.6617 PMC297895

[B18] Hakala M. Tuominen I. Keränen M. Tyystjärvi T. Tyystjärvi E. (2005). Evidence for the role of the oxygen-evolving manganese complex in photoinhibition of photosystem II. *Biochimica Biophys. Acta Bioenerget.* 1706 68–80. 10.1016/j.bbabio.2004.09.001 15620366

[B19] Handschumacher R. E. Harding M. W. Rice J. Drugge R. J. Speicher D. W. (1984). Cyclophilin: a specific cytosolic binding protein for cyclosporin A. *Science* 226 544–547. 10.1126/science.6238408 6238408

[B20] Hashimoto K. Kudla J. (2011). Calcium decoding mechanisms in plants. *Biochimie* 93 2054–2059. 10.1016/j.biochi.2011.05.019 21658427

[B21] He P. Shan L. Sheen J. (2007). The use of protoplasts to study innate immune responses. *Methods Mol. Biol.* 354 1–9. 17172739 10.1385/1-59259-966-4:1

[B22] He Z. Li L. Luan S. (2004). Immunophilins and parvulins. Superfamily of peptidyl prolyl isomerases in *Arabidopsis*. *Plant Physiol.* 134 1248–1267. 10.1104/pp.103.031005 15047905 PMC419802

[B23] Herbstová M. Tietz S. Kinzel C. Turkina M. V. Kirchhoff H. (2012). Architectural switch in plant photosynthetic membranes induced by light stress. *Proc. Natl. Acad. Sci. U.S.A.* 109 20130–20135. 10.1073/pnas.1214265109 23169624 PMC3523818

[B24] Jegerschoeld C. Virgin I. Styring S. (1990). Light-dependent degradation of the D1 protein in photosystem II is accelerated after inhibition of the water splitting reaction. *Biochemistry* 29 6179–6186. 10.1021/bi00478a010 2207066

[B25] Koivuniemi A. Aro E. M. Andersson B. (1995). Degradation of the D1-and D2-proteins of photosystem II in higher plants is regulated by reversible phosphorylation. *Biochemistry* 34 16022–16029. 10.1021/bi00049a016 8519758

[B26] Kress W. J. Erickson D. L. (2007). A two-locus global DNA barcode for land plants: the coding rbcL gene complements the non-coding trnH-psbA spacer region. *PLoS ONE* 2:e508 10.1371/journal.pone.0000508 PMC1876818 17551588

[B27] Li Z. Yue H. Xing D. (2012). MAP Kinase 6-mediated activation of vacuolar processing enzyme modulates heat shock-induced programmed cell death in *Arabidopsis*. *New Phytol.* 195 85–96. 10.1111/j.1469-8137.2012.04131.x 22497243

[B28] Lichtenthaler H. K. (1987). Chlorophyls and carotenoids: pigments of photosynthetic biomembranes. *Methods Enzimol.* 148 350–382. 10.1016/0076-6879(87)48036-1

[B29] Liu Y. Ren D. Pike S. Pallardy S. Gassmann W. Zhang S. (2007). Chloroplast-generated reactive oxygen species are involved in hypersensitive response-like cell death mediated by a mitogen-activated protein kinase cascade. *Plant J.* 51 941–954. 10.1111/j.1365-313X.2007.03191.x 17651371

[B30] Lundin B. Nurmi M. Rojas-Stuetz M. Aro E. M. Adamska I. Spetea C. (2008). Towards understanding the functional difference between the two PsbO isoforms in *Arabidopsis thaliana*—insights from phenotypic analyses of PsbO knockout mutants. *Photosynth. Res.* 98 405–414. 10.1007/s11120-008-9325-y 18709442

[B31] Lundin B. Thuswaldner S. Shutova T. Eshaghi S. Samuelsson G. Barber J. (2007a). Subsequent events to GTP binding by the plant PsbO protein: structural changes. GTP hydrolysis and dissociation from the photosystem II complex. *Biochim. Biophys. Acta Bioenerg.* 1767 500–508. 10.1016/j.bbabio.2006.10.009 17223069

[B32] Lundin B. Hansson M. Schoefs B. Vener A. V. Spetea C. (2007b). The *Arabidopsis* PsbO2 protein regulates dephosphorylation and turnover of the photosystem II reaction centre D1 protein. *Plant J.* 49 528–539. 10.1111/j.1365-313X.2006.02976.x 17217465

[B33] Mattoo A. K. Pick U. Hoffman-Falk H. Edelman M. (1981). The rapidly metabolized 32,000-dalton polypeptide of the chloroplast is the “proteinaceous shield” regulating photosystem II electron transport and mediating diuron herbicide sensitivity. *Proc. Natl. Acad. Sci. U.S.A.* 78 1572–1576. 10.1073/pnas.78.3.1572 6940173 PMC319173

[B34] Miki D. Itoh R. Shimamoto K. (2005). RNA silencing of single and multiple members in a gene family of rice. *Plant Physiol.* 138 1903–1913. 10.1104/pp.105.063933 16172097 PMC1183382

[B35] Mori I. C. Schroeder J. I. (2004). Reactive oxygen species activation of plant Ca2^+^ channels. A signaling mechanism in polar growth, hormone transduction, stress signaling, and hypothetically mechanotransduction. *Plant Physiol.* 135 702–708. 10.1104/pp.104.042069 15208417 PMC514107

[B36] Nelson N. Yocum C. F. (2006). Structure and function of photosystems I and II. *Annu. Rev. Plant Biol.* 57 521–565. 10.1146/annurev.arplant.57.032905.105350 16669773

[B37] Ozgur R. Turkan I. Uzilday B. Sekmen A. H. (2014). Endoplasmic reticulum stress triggers ROS signalling, changes the redox state, and regulates the antioxidant defence of *Arabidopsis thaliana*. *J. Exp. Bot.* 65 1377–1390. 10.1093/jxb/eru034 24558072 PMC3969530

[B38] Pottosin I. Velarde-Buendía A. M. Bose J. Zepeda-Jazo I. Shabala S. Dobrovinskaya O. (2014). Cross-talk between reactive oxygen species and polyamines in regulation of ion transport across the plasma membrane: implications for plant adaptive responses. *J. Exp. Bot.* 65 1271–1283. 10.1093/jxb/ert423 24465010

[B39] Prasil O. Adir N. Ohad I. (1992). Dynamics of photosystem II: mechanism of photoinhibition and recovery process. *Top. Photosynth.* 11 295–348.

[B40] Ralph P. J. Schreiber U. Gademann R. Kühl M. Larkum A. W. (2005). Coral photobiology studied with a new imaging pulse amplitude modulated fluorometer. *J. Phycol.* 41 335–342. 10.1111/j.1529-8817.2005.04034.x

[B41] Rintamäki E. Kettunen R. Aro E. M. (1996). Differential D1 dephosphorylation in functional and photodamaged photosystem II centers Dephosphorylation is a prerequisite for degradation of damaged D1. *J. Biol. Chem.* 271 14870–14875. 10.1074/jbc.271.25.14870 8663006

[B42] Rintamäki E. Kettunen R. Tyystjärvi E. Aro E. M. (1995a). Light-dependent phosphorylation of D1 reaction centre protein of photosystem II: hypothesis for the functional role in vivo. *Physiol. Plant.* 93 191–195. 10.1034/j.1399-3054.1995.930127.x

[B43] Rintamäki E. Salo R. Lehtonen E. Aro E. M. (1995b). Regulation of D1-protein degradation during photoinhibition of photosystem II in vivo: phosphorylation of the D1 protein in various plant groups. *Planta* 195 379–386. 10.1007/BF00202595

[B44] Robinson H. H. Sharp R. R. Yocum C. F. (1981). Effect of manganese on the nuclear magnetic relaxivity of water protons in chloroplast suspensions. *Biochem. Biophys. Res. Commun.* 93 755–761. 10.1016/0006-291X(80)91141-9 6770854

[B45] Rokka A. Aro E. M. Herrmann R. G. Andersson B. Vener A. V. (2000). Dephosphorylation of photosystem II reaction center proteins in plant photosynthetic membranes as an immediate response to abrupt elevation of temperature. *Plant Physiol.* 123 1525–1536. 10.1104/pp.123.4.1525 10938368 PMC59108

[B46] Romano P. Gray J. Horton P. Luan S. (2005). Plant immunophilins: functional versatility beyond protein maturation. *New Phytol.* 166 753–769. 10.1111/j.1469-8137.2005.01373.x 15869639

[B47] Romeis T. Ludwig A. A. Martin R. Jones J. D. (2001). Calcium-dependent protein kinases play an essential role in a plant defence response. *EMBO J.* 20 5556–5567. 10.1093/emboj/20.20.5556 11597999 PMC125278

[B48] Schreiber S. L. (1991). Chemistry and biology of the immunophilins and their immunosuppressive ligands. *Science* 251 283–287. 10.1126/science.1702904 1702904

[B49] Shen Y. Lin H. Y. Huang Z. F. Chen D. F. Li B. H. Xie S. S. (2011). Indirect imaging of singlet oxygen generation from a single cell. *Laser Phys. Lett.* 8 232 10.1002/lapl.201010113

[B50] Shi L. X. Schröder W. P. (2004). The low molecular mass subunits of the photosynthetic supracomplex, photosystem II. *Biochim. Biophys. Acta Bioenerget.* 1608 75–96. 10.1016/j.bbabio.2003.12.004 14871485

[B51] Sirpiö S. Khrouchtchova A. Allahverdiyeva Y. Hansson M. Fristedt R. Vener A. V. (2008). AtCYP38 ensures early biogenesis, correct assembly and sustenance of photosystem II. *Plant J.* 55 639–651. 10.1111/j.1365-313X.2008.03532.x 18445132

[B52] Spetea C. Hundal T. Lundin B. Heddad M. Adamska I. Andersson B. (2004). Multiple evidence for nucleotide metabolism in the chloroplast thylakoid lumen. *Proc. Natl. Acad. Sci. U.S.A.* 101 1409–1414. 10.1073/pnas.0308164100 14736920 PMC337066

[B53] Steinmann B. Bruckner P. Superti-Furga A. (1991). Cyclosporin A slows collagen triple-helix formation in vivo: indirect evidence for a physiologic role of peptidyl-prolyl cis-trans-isomerase. *J. Biol. Chem.* 266 1299–1303. 1985948

[B54] Tikkanen M. Nurmi M. Kangasjärvi S. Aro E. M. (2008). Core protein phosphorylation facilitates the repair of photodamaged photosystem II at high light. *Biochim. Biophys. Acta* 1777 1432–1437. 10.1016/j.bbabio.2008.08.004 18774768

[B55] Vasudevan D. Fu A. Luan S. Swaminathan K. (2012). Crystal structure of *Arabidopsis* cyclophilin38 reveals a previously uncharacterized immunophilin fold and a possible autoinhibitory mechanism. *Plant Cell* 24 2666–2674. 10.1105/tpc.111.093781 22706283 PMC3406915

[B56] Vener A. V. Rokka A. Fulgosi H. Andersson B. Herrmann R. G. (1999). A cyclophilin-regulated PP2A-like protein phosphatase in thylakoid membranes of plant chloroplasts. *Biochemistry* 38 14955–14965. 10.1021/bi990971v 10555977

[B57] Yue H. Nie S. Xing D. (2012). Over-expression of *Arabidopsis* Bax inhibitor-1 delays methyl jasmonate-induced leaf senescence by suppressing the activation of MAP kinase 6. *J. Exp. Bot.* 63 4463–4474. 10.1093/jxb/ers122 22563118

[B58] Zhang W. H. Rengel Z. Kuo J. (1998). Determination of intracellular Ca2^+^ in cells of intact wheat roots: loading of acetoxymethyl ester of Fluo-3 under low temperature. *Plant J.* 15 147–151. 10.1046/j.1365-313X.1998.00188.x

[B59] Zhao Y. Zhou J. Xing D. (2014). Phytochrome B-mediated activation of lipoxygenase modulates an excess red light-induced defence response in *Arabidopsis*. *J. Exp. Bot.* 65 4907–4918. 10.1093/jxb/eru247 24916071 PMC4144769

[B60] Zhou J. Sun A. Xing D. (2013). Modulation of cellular redox status by thiamine-activated NADPH oxidase confers *Arabidopsis* resistance to Sclerotinia *sclerotiorum*. *J. Exp. Bot.* 64 3261–3272. 10.1093/jxb/ert166 23814275

