# Supplementary material for: ROS-mediated enhanced transcription of CYP38 promotes the plant tolerance to high light stress by suppressing GTPase activation of PsbO2
Source: Front Plant Sci. 2015 Sep 29;6:777. doi: 10.3389/fpls.2015.00777 (PMC4586435; doi:10.3389/fpls.2015.00777)
Supplement: Supplementary file 1 [file Presentation_1.PDF]

Supplementary material

Table 1. Primers for several genes.

**Gene name**	**Primer**
CYP38	U: GAATGACTCAGGATCAAGCCAAG<br>D: CCAAAGACAGCGTAACGACCA
PsbO1	U: ACCCTTCGAGGTTGCTTCAG<br>D: GGGTCCAAGGAAGAGCC
PsbO2	U: ACTGGCAAGAAGTTCTGCTTCGAGCCC<br>D: TTCCGTCTGAACCAACCTCG
CaM3	U: CGTACCCGATAAATACGGTTG<br>D: GACCTAATTTGCATTTCACAAAACC
Actin	U: AACGATTCCTGGACCTGCCTCAATCATACTC<br>D: AGAGATTCAGATGCCCAGAAGTCTTGTTCC
UBQ	U: CCAGGACAAGATGATCTGCC<br>D: AAGAAGCTGAAGCATCCAGC
Rbcl	U: ATGTCACCACAAACAGAGACTAAAGC<br>D: CTTCTGCTACAAATAAGAATCGATCTC

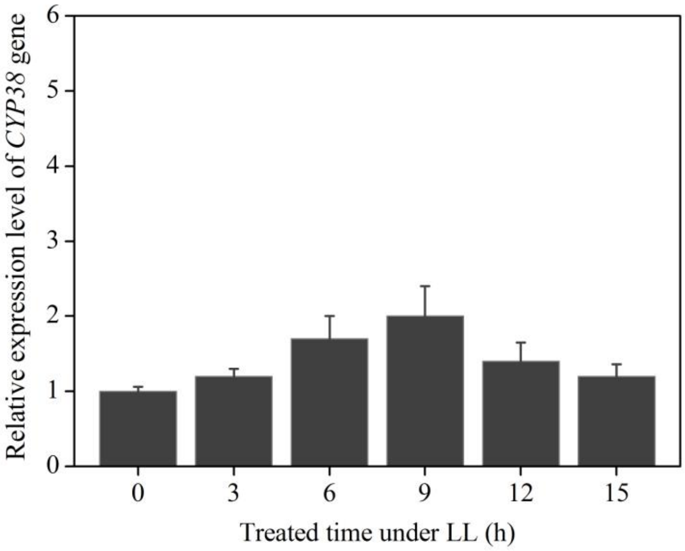

Figure S1

Supplementary Figure 1 Changes of *CYP38* gene expression after low light (LL) treatment in WT plants. qRT-PCR analysis of the expression of *CYP38* gene during 15 h in the LL-treated (50 μmol photons $m^{-2} s^{-1}$) plants WT. For qRT-PCR analysis, total RNAs were extracted from the LL-treated plants at indicated times. Error bars indicate SD values for three replicates.

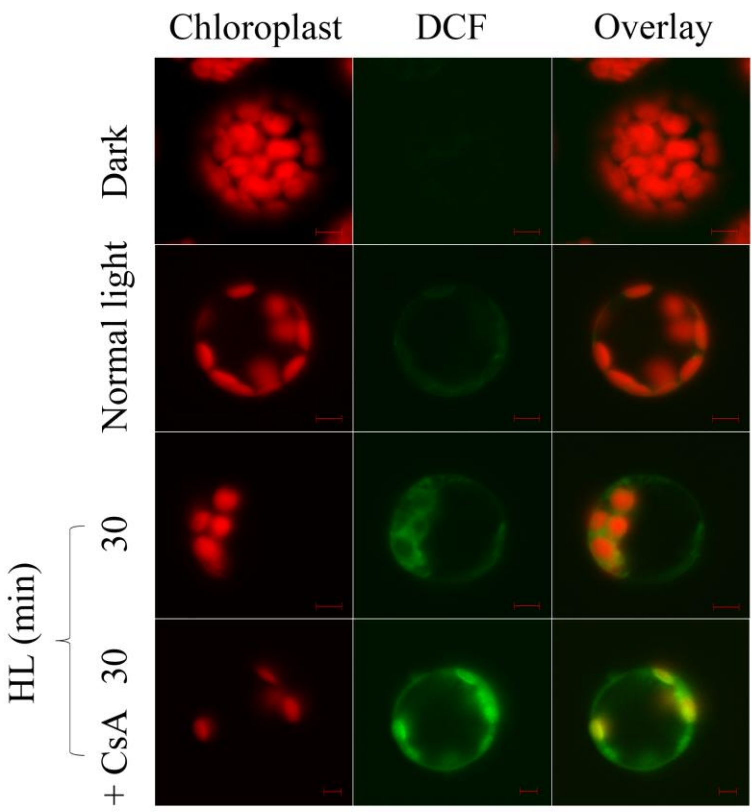

Figure S2

Supplementary Figure 2 Effect of CsA on sub-cellular ROS production after HL treatment. Protoplasts from WT plants were pre-incubated with or without CsA, then incubated with H_2DCFDA for 30 min at room temperature, and observed using LCSM. Protoplasts kept in dark as a control. Chloroplast autofluorescence is false colored red, and DCF fluorescence is false colored green. Scale bars =10 μm.

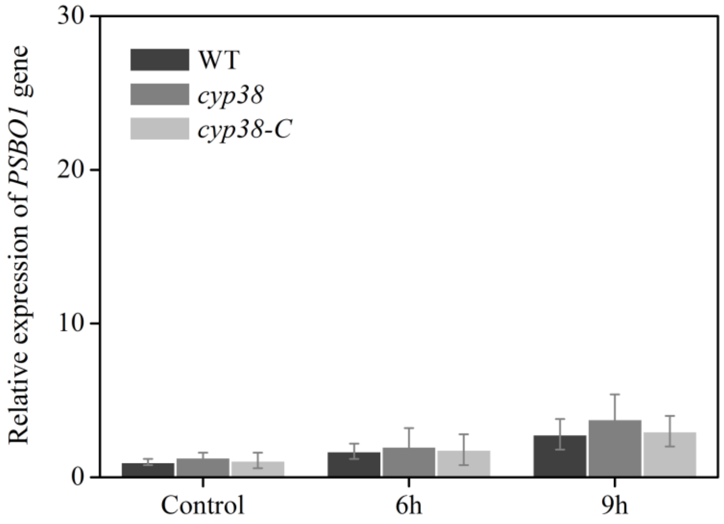

Figure S3

Supplementary Figure 3 Changes of *PSBO1* gene expression in different type plants after HL treatment. qRT-PCR analysis of the expression of *PSBO1* gene in the HL-treated plants of WT, *cyp38* and *cyp38-C* with or without HL treatment. For qRT-PCR analysis, total RNAs were extracted from the HL-treated detached leaves at indicated times. Error bars indicate SD values for three replicates.

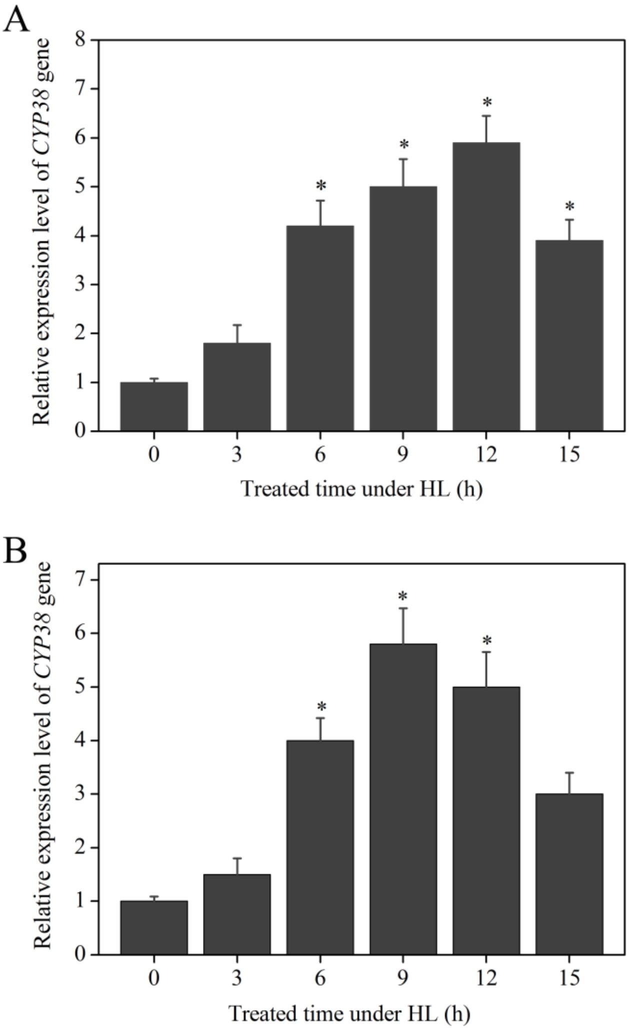

Figure S4

Supplementary Figure 4 Changes of CYP38 gene expression with different genes as the control groups after HL treatment. (A) *Rbcl* gene as the control group. (b) *UBQ* gene as the control group. For qRT-PCR analysis, total RNAs were extracted from the HL-treated detached leaves at indicated times. Error bars indicate SD values for three replicates. Asterisks (*) indicate a significant difference from the control at * $P < 0.05$.
